# Prehospital Cardiopulmonary Resuscitation in Patients with Suspected Severe Traumatic Brain Injury: A BRAIN PROTECT Sub-Analysis

**DOI:** 10.3390/jcm15030934

**Published:** 2026-01-23

**Authors:** Floor J. Mansvelder, Elise Beijer, Anthony R. Absalom, Frank W. Bloemers, Dennis Den Hartog, Nico Hoogerwerf, Esther M. M. Van Lieshout, Stephan A. Loer, Joukje van der Naalt, Lothar A. Schwarte, Sebastiaan M. Bossers, Patrick Schober

**Affiliations:** 1 Department of Anesthesiology, Amsterdam University Medical Center, Vrije Universiteit Amsterdam, de Boelelaan 1117, 1081 HV Amsterdam, The Netherlands; 2 Department of Surgery, Amsterdam University Medical Center, Vrije Universiteit Amsterdam, de Boelelaan 1117, 1081 HV Amsterdam, The Netherlands; 3 Laboratory of Experimental Intensive Care and Anesthesiology, Amsterdam University Medical Center, University of Amsterdam, Meibergdreef 9, 1105 AZ Amsterdam, The Netherlands; 4 Department of Anesthesiology, University Medical Center Groningen, University of Groningen, Hanzeplein 1, 9713 GZ Groningen, The Netherlands; 5 Trauma Research Unit, Department of Surgery, Erasmus MC, University Medical Center Rotterdam, Dr. Molewaterplein 40, 3015 GD Rotterdam, The Netherlands; 6 Department of Anesthesiology, Radboud University Medical Center, Geert Grooteplein Zuid 10, 6525 GA Nijmegen, The Netherlands; 7 Helicopter Emergency Medical Service Lifeliner 3, Zeelandsedijk 10, 5408 SM Volkel, The Netherlands; 8 Department of Neurology, University Medical Center Groningen, Hanzeplein 1, 9713 GZ Groningen, The Netherlands; 9 Helicopter Emergency Medical Service Lifeliner 1, Hornweg 24, 1045 AR Amsterdam, The Netherlands

**Keywords:** severe traumatic brain injury, neurotrauma, prehospital care, cardiopulmonary resuscitation, helicopter emergency medical services, mortality, survival

## Abstract

**Background/Objectives**: Severe traumatic brain injury (TBI) carries high mortality, and outcomes are particularly poor when prehospital cardiopulmonary resuscitation (CPR) is required. Because these patients are often excluded from research, epidemiological data and prognostic insights are limited. This study aimed to describe characteristics and outcomes of patients with suspected severe TBI who received prehospital CPR. **Methods**: We performed a sub-analysis of the prospectively collected multicenter BRAIN-PROTECT registry, including all patients with suspected severe TBI who underwent prehospital CPR and were transported to a participating trauma center. **Results**: A total of 256 patients with suspected severe TBI who received prehospital CPR were included. Early mortality was high, with 22.6% declared dead in the emergency department and an additional 28.9% within 24 h, resulting in 48.5% 24 h survival. Thirty-day mortality was 79.9%. Among survivors, 45.7% achieved moderate disability or good recovery at discharge. Outcomes, 30-day mortality, and neurological status at discharge did not differ between isolated and non-isolated TBI. Characteristics often seen in survivors included shockable initial rhythm, reactive pupils, and lack of anisocoria. All patients without prehospital return of spontaneous circulation died. **Conclusions**: Although overall 30-day mortality was high, survival among patients for whom resuscitation was attempted and who reached hospital care was not negligible, and a substantial proportion of the survivors achieved moderate to good neurological recovery. Prehospital ROSC and shockable rhythms were associated with better outcomes, suggesting that resuscitation may be valuable and warranted in selected patients with potentially reversible conditions. Further studies are needed to better define prognostic factors and guide management in this highly vulnerable population.

## 1. Introduction

Severe traumatic brain injury (TBI) is a major global health problem, associated with high mortality and poor functional outcomes [1,2,3,4,5]. After the first hit of traumatic injury, preventing secondary brain injury becomes the primary focus of early care, starting in the prehospital setting [6,7,8]. Key priorities include preventing hypoxia, avoiding hypotension, maintaining normocapnia, and addressing other major injuries and issues such as major hemorrhage [9,10,11,12]. Failure to achieve these physiological targets is strongly associated with worse outcomes.

Prehospital cardiopulmonary resuscitation (CPR) in patients with severe TBI represents an even more critical challenge. From a pathophysiological perspective, the need for CPR, which involves an additional period of low-flow or no-flow cerebral perfusion on top of the primary brain injury, substantially increases the risk of secondary brain injury [13].

Despite their clinical relevance, patients who receive prehospital CPR are frequently excluded from TBI research because including this subgroup, with its overwhelmingly high mortality rate, introduces significant heterogeneity that can confound analyses aimed at evaluating standard TBI care [14,15]. Conversely, studies on CPR often exclude trauma patients altogether, and research on traumatic cardiac arrest typically does not distinguish between TBI and non-TBI causes [16,17,18]. Consequently, TBI patients requiring CPR remain only sparsely described in the literature. In clinical practice, however, these cases do occur, and clinicians are often faced with uncertainty regarding prognosis and appropriate management strategies.

Against this background, current trauma and neurotrauma guidelines offer no specific recommendations for the management of this specific group of patients with suspected severe TBI who undergo prehospital CPR [19,20]. As a result, epidemiological data and patient outcome estimates are lacking, and it is unclear to what extent historically poor results are truly generalizable. Moreover, possible differences in outcomes between isolated TBI CPR cases and patients with TBI and additional extracranial injuries, and the implications for survival and neurological recovery, are underreported.

To address this knowledge gap, this study aims to describe the prehospital characteristics and in-hospital outcomes of patients with suspected severe TBI who receive prehospital CPR. The overarching aim was to explore whether survival and recovery are uniformly poor, suggesting that continuation of aggressive treatment may be futile, or whether specific patient subgroups demonstrate sufficient potential for meaningful recovery to warrant ongoing care.

## 2. Materials and Methods

### 2.1. Study Design, Setting, Participants

This study is a sub-analysis of prospectively collected data from the BRAIN-PROTECT (Brain Injury; Prehospital Registry of Outcomes, Treatments and Epidemiology of Cerebral Trauma) study, a multicenter observational cohort study aimed at understanding the prehospital management of patients with severe TBI in the Netherlands [21]. Data were collected between February 2012 and December 2017. Inclusion took place when a patient with suspected severe TBI was treated by a Helicopter Emergency Medical Services (HEMS) team. In The Netherlands, suspected severe traumatic brain injury is a primary dispatch criterion for HEMS activation [22]. This suspicion is based on a Glasgow Coma Scale (GCS) score of 8 or lower in combination with a trauma mechanism or clinical findings suggestive of TBI. The study protocol was reviewed by the Medical Research Ethics Committees of Amsterdam University Medical Center (location Vrije Universiteit Medical Center) and Erasmus Medical Center in Rotterdam. These committees determined that the research did not fall under the Dutch Medical Research Involving Human Subjects Act and the requirement for obtaining informed consent was waived [23]. Reporting of the study followed the STROBE (Strengthening the Reporting of Observational Studies in Epidemiology) guidelines [24].

### 2.2. Data Collection and Outcome Measures

For this study, we included and analyzed all patients with suspected severe TBI who underwent prehospital CPR and were transported to a participating trauma center. Patients who were declared dead on the scene were not included. Collected data included demographic characteristics, medication use, American Society of Anesthesiologists (ASA) classification, distance to hospital, duration of CPR, presence of shockable or non-shockable rhythms, prehospital Glasgow Coma Scale (GCS) score, and Injury Severity Score (ISS). Furthermore, data were collected on dispatch category, defined as primary dispatch when Helicopter Emergency Medical Services (HEMS) were activated directly at the time of the emergency call, and secondary dispatch when HEMS was requested by the ambulance team on scene due to clinical deterioration or reassessment of injury severity. The primary outcome of the study was 30-day mortality. Secondary outcomes included mortality in the emergency department, 24 h mortality, neurological status at discharge assessed by the Glasgow Outcome Scale (GOS), length of hospital stay, and length of ICU stay.

### 2.3. Statistical Analyses

Data were analyzed using Stata 18 (StataCorp, College Station, TX, USA). The previously published BRAIN-PROTECT study protocol [23] includes a detailed statistical analysis plan and a power calculation. Data distribution was evaluated using histograms, Shapiro–Wilk tests, and quantile-quantile plots. Continuous variables are presented as means ± standard deviation or medians [25th, 75th percentile], while categorical variables are reported as counts and percentages. For the comparison of two groups, appropriate statistical tests were applied based on variable type and distribution: continuous variables were compared using Student’s *t*-tests or Mann–Whitney U tests, and dichotomous variables were compared using χ^2^ tests or Fisher’s exact tests.

Subgroup analyses were performed in patients with confirmed TBI (head Abbreviated Injury Scale score of 3 or higher) and, separately, in patients with isolated TBI (head Abbreviated Injury Scale score of 3 or higher, scores for all other Abbreviated Injury Scales of 2 or lower) compared with those with non-isolated TBI. All analyses were conducted using complete cases only. Given the substantial proportion of missing data across several key variables, including CPR characteristics and injury severity measures, no imputation techniques were applied. Analyses were therefore explicitly designed to be descriptive and exploratory in nature rather than inferential. Denominators for each variable and analysis are reported throughout the tables to ensure transparency regarding data completeness.

## 3. Results

### 3.1. Patient Demographics and Injury Characteristics

**Figure 1:**
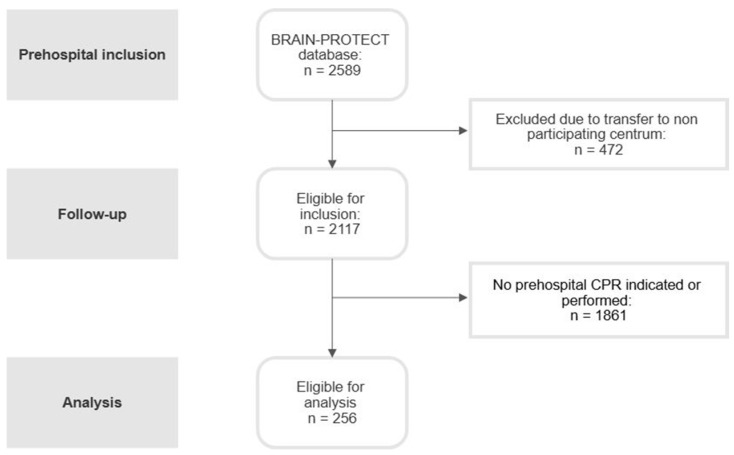
**Flowchart of included TBI patients requiring prehospital CPR.** Abbreviations: CPR, cardiopulmonary resuscitation.

A total of 2589 patients were registered in the BRAIN-PROTECT database. After excluding 472 patients who were transferred to non-participating centers, 2117 patients were eligible for inclusion. Of these, 1861 patients were excluded because they either did not require prehospital CPR or, in cases of cardiac arrest on scene, because CPR was not initiated or was terminated early. The final study cohort included in the analysis consisted of 256 patients who underwent prehospital CPR and were transported to a participating trauma center (Figure 1).

Patient characteristics are summarized in Table 1. The median age of included patients was 46.0 years (IQR 25–66 years), and 72.3% of patients were male. The initial on-scene neurological status was profoundly impaired, with a median prehospital GCS of 3 (IQR 3–3). The median ISS was 34 (IQR 25–43), which reflects a severely traumatized patient population. Falls from height (26.2%) and traffic-related mechanisms (64.3%) were the predominant causes of injury, and most incidents occurred on the road (63.8%). Nearly all injuries were blunt in nature (97.2%), with only a very small proportion of penetrating trauma. Confirmed TBI was present in 92.9% of patients, and 23.2% had isolated TBI. Major prehospital hemorrhage (estimated >1000 mL) was present in 44.6% of included patients. A detailed overview of missing data for all variables included in the analysis is provided in Supplementary Table S1.

### 3.2. Clinical Outcomes

Early outcomes were poor: 22.6% were declared dead in the emergency department and an additional 28.9% within the first 24 h, resulting in 48.5% survival beyond 24 h (Table 1). At 30 days, mortality reached 79.9%, resulting in 48 surviving patients (20.1%). In terms of functional outcome, around 45.7% of patients alive at discharge showed good recovery or only moderate disability, while the other half showed severe disability or a vegetative state.

### 3.3. Patient Characteristics of Survivors and Non-Survivors

Patient characteristics stratified by 30-day survival are presented in Table 2. Survivors and non-survivors did not differ in age or sex distribution. However, survivors had slightly higher initial GCS values (*p* < 0.001) and lower injury severity (ISS median 29 vs. 34, *p* = 0.024). Injury mechanism and location did not significantly differ between groups. Confirmed TBI was more common among non-survivors (95.6% vs. 80.6%, *p* = 0.003), and major hemorrhage occurred significantly more often in non-survivors (55.0% vs. 14.7%, *p* < 0.001).

### 3.4. CPR Specifics

Prehospital CPR characteristics are shown in Table 3. Arrests were witnessed at similar rates in both groups. Although not statistically significant, survivors more often had bystander-initiated CPR in our sample of patients, while non-survivors more frequently received no bystander CPR. AED deployment did not differ between groups. Etiology of arrest differed significantly (*p* = 0.040): cardiac or submersion-related causes were more frequent in survivors, whereas traumatic arrest predominated among non-survivors. CPR Initial rhythm showed the strongest association with survival: 35.3% of survivors presented with VF (Ventricular Fibrillation) compared with 5.2% of non-survivors (*p* < 0.001). Moreover, when grouped as shockable versus non-shockable rhythms, 12.5% of survivors had a shockable rhythm versus only 4.7% of non-survivors (*p* < 0.001). A substantial proportion of patients had no documented initial rhythm during resuscitation. Survival differed across rhythm categories, with the highest survival observed among patients with shockable rhythms, the lowest survival among those with non-shockable rhythms, and intermediate survival among patients with unknown rhythm documentation. Early ROSC (<20 min) was also significantly more common in survivors (100% vs. 79%, *p* = 0.009).

### 3.5. Prehospital Clinical Findings and Interventions

Prehospital clinical parameters and interventions are listed in Table 4. Pupillary reactivity differed substantially between groups: 41.0% of survivors had reactive pupils compared with 12.4% of non-survivors (*p* < 0.001). Anisocoria did not differ between groups. Airway management was performed in nearly all patients, predominantly via endotracheal intubation. Although airway management was statistically more common in non-survivors (100%) than survivors (97%) (*p* = 0.047), the absolute difference was small. Therefore, despite reaching statistical significance, the clinical relevance of this finding is limited. Advanced procedures such as thoracostomy, needle decompression, and chest tube placement were infrequent and showed no significant differences. Transport mode (helicopter vs. ground transport) and dispatch category (primary vs. secondary) were also comparable. Air distance from the scene to the hospital did not differ between survivors and non-survivors.

### 3.6. Subgroup Analysis: Isolated TBI

Mortality and clinical outcomes for patients with isolated versus non-isolated TBI are summarized in Table 5. Early clinical outcomes showed no significant differences between groups (*p* = 0.120). Thirty-day mortality was nearly identical (80.8% vs. 80.6%, *p* = 0.913). Functional outcomes at discharge were similarly distributed between isolated and non-isolated TBI (*p* = 0.184).

### 3.7. Subgroup Analysis: Confirmed TBI

In total, 152 patients had confirmed TBI, 12 had non-confirmed TBI, and for 87 patients, information on TBI confirmation was missing. Mortality at 30 days was significantly higher among patients with confirmed TBI compared with those without confirmed TBI (132 patients, 84.7%, versus 6 patients, 50.0%). Early outcomes, including death in the emergency department, death within 24 h after admission, and survival beyond 24 h, did not differ significantly between groups. Neurological outcome at discharge, assessed by the GOS, was significantly worse in patients with confirmed TBI (*p* < 0.001).

### 3.8. Subgroup Analysis: Surviving Patients with Good Recovery at Discharge

Thirteen patients achieved a good recovery at discharge (Table 6). These patients were not limited to young age/certain age groups (17–74 years), certain types of mechanism of injury, or specific injury severity (ISS 8–41). Although most presented with a GCS of 3, all 13 achieved prehospital ROSC, and most had shockable rhythms such as VF. Anisocoria was absent in nearly all cases.

## 4. Discussion

### 4.1. Key Findings

This study examined a scarcely described, clinically complex and rare population: patients with severe TBI for whom CPR in the prehospital setting in the Netherlands was initiated and who subsequently entered hospital-based care. The main descriptive finding of this study is that survival in this specific patient group, although limited, is not negligible. Mortality at 24 h was 51.5%, increasing to 79.9 at 30 days. However, the remaining 20.1% of patients who survived demonstrated that favorable outcomes are indeed possible. Notably, around half of these 30-day survivors achieved good to moderate neurological functional recovery at discharge.

Importantly, these results should be interpreted as conditional on resuscitation being attempted and on patients reaching hospital care; they therefore do not imply uniformly favorable outcomes after CPR in all TBI-related arrests. Rather, they indicate that once ROSC is achieved and hospital care is reached, the probability of survival and meaningful neurological recovery may be higher than is often assumed. In daily practice, TBI patients requiring CPR are often considered beyond meaningful recovery, and treatment is frequently regarded as futile. Our findings fill an important knowledge gap and provide a more accurate and nuanced view of the outcome potential for those who survive to definitive hospital care, thereby informing whether continued aggressive treatment may be justified in selected patients.

### 4.2. Interpretation/Clinical Implications

To advance this line of inquiry, it is important to identify and characterize the factors associated with an elevated mortality risk in patients with TBI who require prehospital CPR. Although the mortality rate within the cohort is high (79.9%), comparisons of survivors versus non-survivors yield useful insights. Non-survivors more often showed indicators of greater injury burden and physiological derangement, including higher ISS and a higher prevalence of major hemorrhage. In contrast, survivors more frequently presented with a shockable initial rhythm and all of these patients achieved prehospital ROSC.

The observed 100% mortality among patients who did not achieve prehospital ROSC highlights the extremely narrow therapeutic window in this population and underscores the critical importance of rapid, goal-directed prehospital intervention. In practical terms, these findings suggest that achieving early ROSC is a key prerequisite for any chance of survival, and they support continued efforts toward timely resuscitation to mitigate the cascade of secondary ischemic injury following cardiac arrest, correction of reversible causes, and rapid transport when ROSC is obtained.

Another notable finding is that the proportion of survivors achieving a favorable neurological outcome is comparable to that observed in the broader population of severe TBI patients who did not require CPR [21]. This suggests that, in a selected subset of patients who reach hospital care after successful resuscitation, cardiac arrest does not inevitably translate into universally poor neurological outcomes beyond the already high baseline risk associated with TBI. At the same time, however, it is important to consider that “suspected severe TBI” in the prehospital setting is a working diagnosis, and some patients may have had a primary non-traumatic cause of arrest (e.g., myocardial infarction or another medical event) with secondary trauma as a consequence of collapse. Consistent with this, when restricting the analysis to patients with confirmed clinically relevant head injury severity, survival was markedly lower, and favorable outcomes in the total cohort were to a large extent driven by patients without evidence of severe intracranial injury. Although the number of patients without confirmed TBI was relatively small compared with those with confirmed TBI, this contrast remains clinically informative and underscores the heterogeneity within the population of patients initially classified as having suspected severe TBI.

Importantly, this does not diminish the clinical relevance of our overall findings; rather, it highlights a key real-world challenge: in the prehospital environment, providers can often not reliably distinguish TBI-driven arrests from other causes. This diagnostic uncertainty should therefore be explicitly considered when deciding on initiation or continuation of CPR in patients with suspected TBI. In particular, patients with a shockable rhythm seem to have better outcomes; however, patients with PEA or even asystole have also survived with good recovery, advocating for a carefully selected, aggressive and well-coordinated resuscitation effort in patients with cardiac arrest and (suspected) TBI. Nevertheless, overall prognosis remains poor, and these observations should not be interpreted as justification for indiscriminate resuscitation, but rather as support for a nuanced, physiology-driven approach to decision-making in this high-risk population. Importantly, these observations should also be interpreted as descriptive and hypothesis-generating rather than as evidence of causal or generalizable treatment effects.

Historically, traumatic cardiac arrest was widely regarded as futile, including arrests associated with severe extracranial trauma. In recent years, this view has been challenged by an improved understanding of traumatic arrest pathophysiology and a reprioritization of resuscitation strategies, with emphasis on early identification and correction of reversible causes rather than immediate chest compressions. Structured approaches focusing on hemorrhage control, oxygenation, relief of tension pneumothorax, and management of cardiac tamponade have been associated with improved outcomes in selected patients [25]. The survival observed in the present cohort of patients with TBI and cardiac arrest may, at least in part, reflect the impact of these evolving resuscitation principles within modern prehospital trauma care.

In addition, a specific subgroup of patients with TBI-associated cardiac arrest may be explained by the concept of impact brain apnea [26,27]. In these cases, cardiac arrest occurs shortly following head impact, potentially in the absence of major structural brain injury, and is primarily driven by transient central apnea. When treated early with prompt airway management, oxygenation, and basic CPR to bridge the apneic period, spontaneous circulation and ventilation may be restored before substantial hypoxic brain injury develops. Although the incidence of impact brain apnea cannot be determined in the present cohort, this mechanism may partly explain the favorable outcomes observed in some patients, particularly those with early ROSC and good neurological recovery. This further supports the notion that TBI-associated cardiac arrest does not represent a uniform pathophysiological entity or prognosis.

Given the relatively young age of this patient population and the substantial disability-adjusted life years at stake, the potential for meaningful neurological recovery in a subset of patients underscores the broader societal relevance of this condition. In this context, our findings suggest that continued refinement of prehospital trauma care, including rapid assessment, controlled resuscitation, and timely transport, may influence outcomes in selected patients with severe TBI and cardiac arrest.

### 4.3. Comparison with Literature

Reported survival rates of traumatic cardiac arrest vary widely across studies, ranging from approximately 4% to 40%, depending on inclusion criteria, case mix, and definitions of survival [28,29,30], largely due to differences in study design and case selection [27]. Importantly, many of these studies report overall survival rather than neurologically meaningful recovery, which is substantially less frequent [31].

While the aforementioned literature describes traumatic cardiac arrest in general and does not consistently distinguish between TBI and non-TBI patients, to our knowledge, only one previous study has examined a comparable population of patients with severe TBI requiring prehospital CPR. Zhao et al. [32] analyzed a smaller cohort of 42 patients, reporting a 19% survival rate, with most survivors achieving favorable neurological outcomes. Our findings broadly align with these observations. In our larger national cohort, 20.1% of patients survived to 30 days, approximately half of whom achieved moderate to good neurological recovery at discharge. In both studies, favorable neurological signs, including preserved pupillary reactivity, were associated with survival.

When placed in a broader context beyond trauma, the survival rate observed in our cohort appears comparable to that reported for out-of-hospital cardiac arrest in the general Dutch population. In the Netherlands, survival after EMS-treated OHCA is approximately 23% [33]. However, this comparison should be interpreted cautiously because case mix and inclusion criteria differ substantially. Importantly, the present cohort is conditional on patients reaching hospital care. Nonetheless, the data suggest that outcomes in patients with suspected TBI who require CPR may not be as uniformly poor as traditionally assumed.

### 4.4. Strengths and Limitations

This study has several notable strengths. Leveraging data from a large, prospectively collected multicenter registry allowed us to characterize a rare and critically ill population of patients who underwent prehospital CPR for suspected severe TBI. The availability of detailed prehospital information, alongside outcome data, enabled a comprehensive description of patient characteristics, resuscitation patterns, and clinically relevant prognostic signals.

However, several limitations should be considered. First, the cohort definition introduces inherent selection: the results are conditional on resuscitation being attempted and on patients reaching hospital care; therefore, the findings are not directly generalizable to all patients with suspected TBI-related arrest at the scene.

Second, although most core clinical variables (including age, GCS, ISS, airway management, and mortality outcomes) were largely complete, several CPR-specific and prehospital operational variables exhibited substantial proportions of missing data. This was particularly pronounced for cardiac rhythm during resuscitation, for which nearly half of the cohort lacked documented data. As all analyses were restricted to complete cases for the variables under study, selection bias cannot be excluded, especially in subgroup analyses. The high degree of missingness limits the interpretability of rhythm-related findings. Although shockable rhythms were more frequently observed among survivors, this observation should be interpreted with caution. To improve transparency, patients with undocumented initial rhythm were included as a separate descriptive category, revealing intermediate survival compared with shockable and non-shockable rhythms. However, given the extent of missing data and limited subgroup sizes, formal sensitivity analyses were not performed, as these would have been severely underpowered. All rhythm-related findings are therefore presented descriptively and should not be interpreted as evidence of a causal association.

Third, the extremely high 30-day mortality in this cohort limited the discriminative power of statistical modeling, restricting analyses primarily to descriptive and exploratory interpretations. This limitation, combined with heterogeneous arrest etiology and incomplete data, precluded robust comparative or causal inference.

Fourth, important neurological prognostic factors known to influence outcome after severe traumatic brain injury, such as the presence and extent of diffuse axonal injury, were not consistently available in the dataset and could therefore not be included in the analyses. Diffuse axonal injury is recognized as a key determinant of neurological outcome after TBI, but its reliable assessment requires detailed neuroimaging data that were beyond the scope of the present registry-based study [34].

Fifth, neurological outcome was assessed using the Glasgow Outcome Scale at hospital discharge, which represents an early time point after severe traumatic brain injury and may not reliably reflect long-term functional recovery. Patients may substantially improve or deteriorate in the months following discharge. As long-term follow-up data were not consistently available within the BRAIN-PROTECT registry, the proportion of patients classified as having “good recovery” should be interpreted as early neurological status rather than definitive long-term outcome.

Finally, the subgroup of patients achieving good neurological recovery was small. While clinically informative, these observations should be considered hypothesis-generating and interpreted within the broader context of the study’s descriptive design and methodological constraints. In addition, because this study was conducted within a physician-staffed Helicopter Emergency Medical Services system with advanced on-scene capabilities, the observed outcomes may not be directly generalizable to paramedic-led EMS systems with more limited prehospital treatment options.

## 5. Conclusions

In this study of patients with TBI requiring prehospital CPR, we found that, despite an overall high 30-day mortality rate of approximately 80%, a meaningful proportion (about one in five patients) survived, and roughly half of these survivors achieved moderate to good neurological functional outcomes at hospital discharge. Key favorable signals included early ROSC and the presence of shockable initial rhythms at the scene. These findings suggest that resuscitation efforts may be considered in selected patients with suspected TBI, as outcomes may not be uniformly futile in those who achieve prehospital ROSC, while acknowledging the overall poor prognosis and heterogeneity of this population. Larger prospective studies are needed to better identify which subgroups are most likely to benefit from aggressive resuscitative interventions and to optimize treatment strategies for this vulnerable population.

## Figures and Tables

**Table 1:** Patient characteristics of patients with suspected severe TBI who underwent prehospital CPR.

	All Patients n = 256	Missing (n)
	Median [quartiles]/n (percentage)	
**Age**	46.0 [25–64]	5
**Male sex**	185 (72.3)	0
**First GCS**	3 [3–3]	0
**ISS**	34 [25–43]	50
**Injury Mechanism**		4
Traffic, motor vehicle	38 (15.1)	
Traffic, motorcycle	32 (12.7)	
Traffic, bicycle	55 (21.8)	
Traffic, pedestrian	28 (11.1)	
Traffic, other	9 (3.6)	
Fall from height	66 (26.2)	
Gunshot or stab injury	4 (1.6)	
Other	20 (7.9)	
**Injury Type**		4
Blunt	245 (97.2)	
Penetrating	6 (2.4)	
Combined	1 (0.4)	
**Injury location**		10
Road	172 (67.2)	
Workplace	12 (4.7)	
Home	44 (17.2)	
Sport Facility	4 (1.6)	
Other	14 (5.5)	
**Confirmed TBI**	157 (92.9)	87
**Isolated TBI**	47 (23.2)	53
**Hemorrhage > 1000 mL**	78 (44.6)	81
**Early outcome**		17
Death in emergency department	54 (22.6)	
Additional death within 24 h after admission	69 (28.9)	
Survival > 24 h	116 (48.5)	
**Mortality at 30 days**		17
Alive	48 (20.1)	
Death	191 (79.9)	
**GOS at discharge**		19
Death	191 (80.6)	
Persistent vegetative state	3 (1.27)	
Severe disability	22 (9.28)	
Moderate disability	8 (3.38)	
Good recovery	13 (5.49)	

Abbreviations: GCS, Glasgow Coma Score; ISS, Injury Severity Score; TBI, Traumatic Brain Injury; GOS, Glasgow Outcome Scale.

**Table 2:** Patient characteristics of patients with suspected severe TBI who underwent prehospital CPR. Survivors compared to non-survivors at 30 days.

	Non-Survivors n = 191 (79.9%)	Survivors n = 48 (20.1%)	*p*-Value	Missing (n)
	Median [quartiles]/n (percentage)			
**Age**	47 [25–66]	44.5 [25.5–56.5]	0.436	0
**Male sex**	135 (70.7)	36 (75.0%)	0.553	0
**First GCS**	3 [3–3]	3 [3–3.5]	<0.001	0
**ISS**	34 [25–45]	29 [16.5–34.5]	0.024	25
**Injury Mechanism**			0.114	3
Traffic, motor vehicle	27 (14.3)	8 (17.0)		
Traffic, motorcycle	21 (11.1)	10 (21.3)		
Traffic, bicycle	43 (22. 8)	10 (21.3)		
Traffic, pedestrian	25 (13.2)	2 (4.3)		
Traffic, other	4 (2.1)	4 (8.3)		
Fall from height	51 (27.0)	10 (21.3)		
Gunshot or stab injury	3 (1.6)	1 (2.13)		
Other	15 (7.9)	2 (4.26)		
**Injury Type**			0.856	3
Blunt	182 (96.8)	47 (97.9)		
Penetrating	5 (2.7)	1 (2.1)		
Combined	1 (0.5)	0 (0.0)		
**Injury location**			0.262	10
Road	127 (69.8)	36 (76.6)		
Workplace	8 (4.4)	3 (6.4)		
Home	33 (18.1)	5 (10.6)		
Sport Facility	2 (1.1)	2 (4.3)		
Other	12 (6.6)	1 (2.1)		
**Confirmed TBI**	132 (95.7)	25 (80.7)	0.003	70
**Isolated TBI**	38 (23.3)	9 (22.5)	0.913	34
**Hemorrhage > 1000 mL**	71 (55.0)	5 (14.7)	<0.001	76

Abbreviations: GCS, Glasgow Coma Score; ISS, Injury Severity Score; TBI, traumatic brain injury.

**Table 3:** Specifications for prehospital cardiopulmonary resuscitation in patients with suspected severe TBI.

	All Patients n = 256	Missing Variable Data(n)	Non-Survivors n = 191 (79.9%)	Survivors n = 48 (20.1%)	*p*-Value	Missing Survival Data (n)
	Median [quartiles]/n (percentage)					
**Witnessed arrest**	111 (74.5)	107	92 (75.4)	15 (75.0)	0.969	97
**CPR bystander**		105			0.066	96
Chest compressions and ventilation	67 (44.4)		51 (43.2)	14 (56.0)		
Chest compressions only	30 (19.9)		21 (17.8)	8 (32.0)		
Ventilation only	1 (0.7)		1 (0.9)	0 (0.0)		
None	53 (35.1)		45 (38.1)	3 (12.0)		
**AED bystander**		126			0.430	116
Yes	25 (19.2)		18 (17.5)	5 (25.0)		
No	105 (80.8)		85 (82.5)	15 (75.0)		
**CPR etiology**		115			0.040	43
Cardiac	15 (7.3)		9 (5.5)	5 (15.2)		
Traumatic	184 (89.3)		150 (92.0)	26 (78.8)		
Submersion	3 (1.5)		1 (0.6)	2 (6.1)		
Respiratory other than submersion	1 (0.5)		1 (0.6)	0 (0.0)		
Other	3 (1.5)		2 (1.2)	0 (0.0)		
**CPR First rhythm**					<0.001	106
VF	12 (8.5)		6 (5.2)	6 (35.3)		
VT	1 (0.7)		1 (0.9)	0 (0.0)		
Asystole	57 (40.4)		50 (43.1)	1 (5.9)		
PEA	53 (37.6)		49 (42.2)	4 (23.5)		
Bradycardia in children	2 (1.4)		0 (0.0)	1 (5.9)		
After ROSC	16 (11.4)		10 (8.6)	5 (29.4)		
**Initial rhythm**		120			<0.001	112
Shockable	15 (5.9)		9 (4.7)	6 (12.5)		
Non-shockable	121 (47.3)		107 (45.0)	6 (12.5)		
Unknown rhythm	120 (46.9)		75 (39.3)	36 (75.0)		
Number of defibrillations	1.5 [1–3]		2.5 [1–3]	1 [1–2.5]	0.234	
**Prehospital ROSC (<20 min)**		75			0.009	54
Yes	150 (82.9)		118 (79.2)	27 (100.00)		
No or transport with CPR	31 (17.1)		31 (20.8)	0 (0.0)		

The first *Missing* (*n*) column refers to missing data for the variable of interest. The second *Missing* (*n*) column refers to patients with missing survival outcome data, who were not included in the comparison between non-survivors and survivors. Abbreviations: AED, automated external defibrillator; CPR, cardiopulmonary resuscitation; PEA, pulseless electrical activity; ROSC, return of spontaneous circulation; TBI, Traumatic Brain Injury; VF, ventricular fibrillation; VT, ventricular tachycardia.

**Table 4:** Summary of prehospital clinical findings and interventions, including neurologic assessment, airway management, and treatments provided in the prehospital setting.

	All Patients n = 256	Missing Variable Data(n)	Non-Survivors n = 191 (79.9%)	Survivors n = 48 (20.1%)	*p*-Value	Missing Survival Data (n)
	Median [quartiles]/n (percentage)					
**PEARL**		44			<0.001	39
yes	42 (19.8)		20 (12.42)	16 (41.0)		
no	170 (80.2)		141 (87.58)	23 (59.0.)		
**Anisocoria**		31			0.642	28
yes	52 (23.1)		41 (23.98)	11 (27.5)		
no	173 (76.9)		130 (76.02)	29 (72.5)		
**Airway management**	252 (99.6)	3	189 (100)	47 (97.9)	0.047	0
**Airway device**		2			0.254	1
No airway management	1 (0.4)		0 (0.0)	1 (2.1)		
Endotracheal intubation	238 (93.7)		177 (93.16)	46 (95.8)		
LMA	4 (1.6)		3 (1.58)	1 (2.1)		
Other supraglottic device	3 (1.2)		3 (1.58)	0 (0.0)		
Combitube	1 (0.4)		1 (0.53)	0 (0.0)		
Coniotomy	7 (2.8)		6 (3.16)	0 (0.0)		
**Advanced prehospital manoeuvres**						
Needle thoracostomy on any side	40 (15.6)	0	32 (16.75)	5 (10.4)	0.278	0
Chest tube on any side	16 (6.3)	0	13 (6.81)	1 (2.1)	0.213	0
Pericardial puncture	1 (0.4)	0	1 (0.52)	0 (0.0)	0.615	0
Surgical thoracostomy	21 (8.2)	0	18 (9.42)	2 (4.2)	0.240	0
Thoracotomy	2 (0.8)	0	2 (1.05)	0 (0.0)	0.477	0
**HEMS transport to patient**		1			0.265	1
Helicopter	195 (76.5)		149 (78.42)	34 (70.8)		
HEMS ambulance vehicle	60 (25.5)		41 (21.58)	14 (29.2)		
**Secondary or primary dispatch to scene**		6			0.569	6
Primary dispatch	218 (87.2)		164 (88.17)	40 (85.1)		
Secondary dispatch	32 (12.8)		22 (11.83)	7 (14.9)		
**Air distance from scene to hospital, km**	19.9 [8.6–32.2]	37	19.95 [8.5–31.6]	16.9 [6.0–32.4]	0.507	9

The first *Missing* (*n*) column refers to missing data for the variable of interest. The second *Missing* (*n*) column refers to patients with missing survival outcome data, who were not included in the comparison between non-survivors and survivors. Abbreviations: HEMS, Helicopter Emergency Medical Service; km, kilometer; LMA, laryngeal mask airway; PEARL, pupils equal and reactive to light.

**Table 5:** Subgroup Analysis for patients with Isolated compared to non-isolated Traumatic Brain Injury who underwent prehospital CPR.

	Isolated TBI n = 47 (23.2%)	Non-Isolated TBI n = 156 (76.8%)	*p*-Value	Missing (n)
	Median [quartiles]/n (percentage)			
**Early outcome**			0.120	0
Death in emergency department	15 (31.9)	28 (18.0)		
Additional death within 24 h after admission	13 (27.7)	50 (32.1)		
Survival >24 h	19 (40.4)	78 (50.0)		
**Mortality at 30 days**			0.913	0
Alive	9 (19.2)	31(19.9)		
Death	38 (80.9)	125 (80.1)		
**GOS at discharge**			0.184	1
Death	38 (80.9)	125 (80.7)		
Persistent vegetative state	0 (0.0)	2 (1.3)		
Severe disability	4 (8.5)	16 (10.3)		
Moderate disability	4 (8.5)	3 (1.9)		
Good recovery	1 (2.1)	9 (5.8)		

Abbreviations: CPR, cardiopulmonary resuscitation; GOS, Glasgow Outcome Scale; TBI, traumatic brain injury.

**Table 6:** Overview of CPR-treated patients with suspected severe TBI who achieved a good recovery.

	Age (Years)	ISS	GCS	CPR First Rhythm	Injury Mechanism	Anisocoria	Prehospital ROSC	Craniotomy?
Patient 1 (10)	48	10	4	After ROSC	Motorcycle	No	yes	No
Patient 2 (55)	38	–	5	–	Traffic, other	No	yes	No
Patient 3 (112)	51	–	3	–	Fall from height	No	yes	No
Patient 4 (113)	28	16	3	VF	Motor Vehicle	–	yes	No
Patient 5 (140)	65	–	3	VF	Fall from height	–	yes	No
Patient 6 (147)	17	13	3	PEA	Motor Vehicle	No	yes	No
Patient 7 (176)	54	41	3	VF	Bicycle	–	yes	No
Patient 8 (179)	47	29	3	–	Motorcycle	No	yes	No
Patient 9 (181)	25	34	3	Asystole	Gunshot/stab injury	No	yes	No
Patient 10 (191)	20	10	3	PEA	Fall from height	No	yes	No
Patient 11 (234)	74	16	6	–	Fall from height	No	yes	No
Patient 12 (238)	68	29	3	–	Other	No	yes	No
Patient 13 (251)	45	8	3	–	Pedestrian	No	yes	No

“–“ indicates missing or unknown data. Abbreviations: CPR, cardiopulmonary resuscitation; GCS, Glasgow Coma Scale; ISS, Injury Severity Score; PEA, pulseless electrical activity; ROSC, return of spontaneous circulation; TBI, traumatic brain injury; VF, ventricular fibrillation.

## Data Availability

Partial or complete datasets and the data dictionary are available upon reasonable request to the author, Floor Mansvelder, pending approval by the scientific steering committee.

## Supplementary Materials

The following supporting information can be downloaded at: https://www.mdpi.com/article/10.3390/jcm15030934/s1, Table S1: Summary of missing data.

## Abbreviations

The following abbreviations are used in this manuscript:

AIS	Abbreviated injury scale
ASA	American Society of Anesthesiologists Physical Status
CPR	Cardiopulmonary Resuscitation
ER	Emergency Department
GCS	Glasgow Coma Scale
GOS	Glasgow Outcome Scale
HEMS	Helicopter Emergency Medical Service
ICU	Intensive Care Unit
IQR	Interquartile Range
IRR	Incidence Risk Ratio
ISS	Injury Severity Score
OR	Odds Ratio
PEA	Pulseless Electrical Activity
ROSC	Return Of Spontaneous Circulation
TBI	Traumatic Brain Injury
VF	Ventricular Fibrillation
VT	Ventricular Tachycardia
