# Supplementary material for: Prehospital Cardiopulmonary Resuscitation in Patients with Suspected Severe Traumatic Brain Injury: A BRAIN PROTECT Sub-Analysis
Source: J Clin Med. 2026 Jan 23;15(3):934. doi: 10.3390/jcm15030934 (PMC12898378; doi:10.3390/jcm15030934)
Supplement: Supplementary file 1 [file jcm-15-00934-s001.zip › jcm-4083294-supplementary.pdf]

Supplementary Table S1. Summary of missing data.

Variables	Number of patient N = 256	Missing (n)	Missing (%)
**Patient characteristics**			
**Age**	251	5	2.0%
**Male sex**	256	0	0.0%
**First GCS**	256	0	0.0%
**ISS**	206	50	24.3%
**Injury mechanism**	252	4	1.6%
**Injury type**	252	4	1.6%
**Injury location**	246	10	3.9%
**Confirmed TBI**	169	87	34.0%
**Isolated TBI**	203	53	26.1%
**Hemorrhage >1000 ml**	175	81	46.3%
**Early outcome**	239	17	6.6%
**Mortality at 30 days**	239	17	6.6%
**GOS at discharge**	237	19	7.4%
**CPR characteristics**			
**Witnessed arrest**	111	145	56.6%
**CPR bystander**	151	105	41.0%
**AED bystander**	130	126	49.2%
**CPR etiology**	141	115	44.9%
**CPR first rhythm**	141	115	44.9%
**Initial rhythm**	136	120	46.9%
**Prehospital ROSC**	181	75	29.3%
**Prehospital findings and interventions**			
**PEARL**	212	44	17.2%
**Anisocoria**	225	31	12.1%
**Airway management**	253	3	1.2%
**Airway device**	254	2	0.8%
**Advanced prehospital manoeuvres**	256	0	0.0%
**Needle thoracostomy**	256	0	0.0%
**Chest tube**	256	0	0.0%
**Pericardial puncture**	256	0	0.0%
**Surgical thoracostomy**	256	0	0.0%
**Thoracotomy**	256	0	0.0%
**HEMS transport to patient**	255	1	0.0%
**Secondary or primary dispatch to scene**	250	6	0.0%
**Air distance from scene to hospital, km**	219	37	0.0%

Abbreviations: AED, automated external defibrillator; CPR, cardiopulmonary resuscitation; GCS, Glasgow Coma Score; GOS, Glasgow Outcome Scale; HEMS, helicopter emergency medical services; ISS, Injury Severity Score; PEARL, pupils equal and reactive to light;ROSC, return of spontaneous circulation; TBI, Traumatic Brain Injury.
